# Oxidative Stress-Related lncRNAs Are Potential Biomarkers for Predicting Prognosis and Immune Responses in Patients With LUAD

**DOI:** 10.3389/fgene.2022.909797

**Published:** 2022-06-08

**Authors:** Xinti Sun, Xingqi Huang, Xiaojuan Sun, Si Chen, Zeyang Zhang, Yao Yu, Peng Zhang

**Affiliations:** ^1^ Department of Thoracic Surgery, Tianjin Medical University General Hospital, Tianjin, China; ^2^ Department of Neurosurgery, Tianjin Medical University General Hospital, Tianjin, China; ^3^ Department of Oncology, Qingdao University Affiliated Hospital, Qingdao, China

**Keywords:** lung adenocarcinoma, lncRNA, oxidative stress, bioinformatics, tumor immune

## Abstract

Lung adenocarcinoma is increasingly harmful to society and individuals as cancer with an inferior prognosis and insensitive to chemotherapy. Previous studies have demonstrated that oxidative stress and lncRNAs play a vital role in many biological processes. Therefore, we explored the role of lncRNAs associated with oxidative stress in the prognosis and survival of LUAD patients. We examined the expression profiles of lncRNAs and oxidative stress genes in this study. A prognosis prediction model and a nomogram were built based on oxidative stress-related lncRNAs. Functional and drug sensitivity analyses were also performed depending on oxidative stress-related lncRNA signature. Moreover, we investigated the relationship between immune response and immunotherapy. The results showed that a risk scoring model based on 16 critical oxidative stress lncRNAs was able to distinguish the clinical status of LUAD and better predict the prognosis and survival. Additionally, the model demonstrated a close correlation with the tumor immune system, and these key lncRNAs also revealed the relationship between LUAD and chemotherapeutic drug sensitivity. Our work aims to provide new perspectives and new ideas for the treatment and management of LUAD.

## Introduction

Lung cancer, the primary malignant tumor, accounts for the main reason for cancer-related deaths worldwide (Schabath and DiGiovanni, 2015; Bray et al., 2018). A large percentage of lung cancers (approximately 85%) are non-small cell carcinomas (NSCLCs), and nearly 60% of patients have metastasized locally or distantly (Tun et al., 2019). The most common type of non-small cell lung cancer is lung adenocarcinoma (LUAD), with its incidence exceeding lung squamous cell carcinoma (Tong et al., 2018). Although clinical outcomes for patients with LUAD have significantly improved because of advances in diagnosis, surgery, radiation therapy, and molecular therapy, LUAD patients still have a relatively low 5-year survival rate (Zhang et al., 2019; Jurisic et al., 2020). The evidence that molecular biomarkers can be used for predictive purposes has been snowballing over the past few years, and these biomarkers have been discovered and applied (Jiao and Wang, 2016).

Oxidative stress is a state where there is an imbalance between the production of reactive oxygen species (ROS) and the effectiveness of antioxidants because of the imbalance between the production of free radicals and the ability to neutralize these oxidative molecules (Brown et al., 2020). Oxidative stress induced by reactive oxygen species (ROS) has become increasingly recognized as having an essential role in cancer development (Hussain et al., 2003). Studies have shown that cancer patients have reduced antioxidant status and elevated levels of oxidative stress even before tumor therapy begins. Furthermore, many biomarkers have been used to understand oxidative stress’ role in cancer pathophysiology (Jelic et al., 2021). An earlier study identified a set of oxidative stress genes implicated in the prognosis and progression of gastric cancer and may be used as potential prognostic and diagnostic biomarkers (Wu Z. et al., 2021).

Long non-coding RNAs (lncRNAs) comprise the majority of non-coding RNAs and represent transcripts with a length greater than 200 nucleotides. Among their functions are chromatin remodeling and transcriptional and post-transcriptional regulation (Kopp and Mendell, 2018). Additionally, lncRNAs are thought to influence tumor cell migration by regulating target genes (Ramilowski et al., 2020). Recently, several studies have demonstrated that immune-related lncRNAs and other lncRNAs may enhance the predictive value of LUAD patients (Sacks et al., 2018; Li et al., 2020). However, oxidative stress-related lncRNA signatures of LUAD have not been widely used.

This is the first bioinformatics study to reveal the association between oxidative stress-related lncRNAs and LUAD. The study utilized TCGA database to obtain the expression profiles of lncRNAs and genes related to oxidative stress. We identified the lncRNAs associated with oxidative stress using Pearson’s correlation analysis. LUAD patients with low risk scores are predicted to have better overall survival using this novel oxidative stress-related lncRNA prognostic model. Furthermore, we detected potential drug candidates aiming for this lncRNA signature associated with oxidative stress based on publicly available drug sensitivity databases. Additionally, we examined the relationship between immunotherapy and patient response. Last, we constructed a nomogram to predict the survival of LUAD patients. Based on the aforementioned studies, we aimed to provide new guidance for the clinical treatment of LUAD and further reveal the mechanism of oxidative stress in LUAD.

## Methods

### Data Acquisition and Preprocessing

All data of LUAD patients (tumor = 535 and normal = 59) were downloaded from TCGA database (http://portal.gdc.cancer.gov/). In total, 807 oxidative stress-related genes were obtained from GeneCards (https://www.genecards.org) with a relevance score ≥7 (Wu Z. et al., 2021). Further preprocessed with the “limma” package in R ((FDR) < 0.05 and |log2 fold change (FC)|≥1), 199 differentially expressed oxidative stress genes were identified. We screened oxidative stress-related lncRNAs using Pearson’s correlation test (Pearson correlation coefficient >0.4, *p* < 0.001), and 3,295 oxidative stress-related lncRNAs were obtained. Volcano maps were created using the R package “ggplot2.” LUAD patients with missing overall survival values and short overall survival values (<30 days) were removed to reduce statistical bias. We obtained 490 samples and divided them into training and testing sets at random. Clinical characteristics were not significantly different between training and testing sets (*p* > 0.05). A total of 246 samples in the training set were used to develop a predictive risk model. The testing set included 244 samples used to validate the established risk model.

### Construction and Validation of the Risk Model

Univariate Cox regression, LASSO regression, and multivariate regression analyses were applied to analyze the oxidative stress-related lncRNAs using R. The risk score is calculated with the formula as follows:

$$Risk\;score=\overset{n}{\underset{k=1}{\sum }}Coef\left(lncRNA\right)\;*\;expr\left(lncRNA^{k}\right),$$

where coef (lncRNA) represents the correlation coefficient between lncRNAs and survival and expr represents the expression of lncRNAs. Patients were divided into high-risk and low-risk groups according to the median risk score.

### Independent Factors and ROC

Univariate Cox and multivariate Cox regression analyses were conducted to verify whether risk scores and clinical characteristics were independent variables, and ROC curves were applied to compare the performance of the various factors in predicting outcomes.

### Survival Analysis and Principal Component Analysis

Kaplan–Meier (K-M) survival analysis determined the overall survival (OS) of patients in subgroups, including low-risk and high-risk groups using the “survival” package in R. Principal component analysis (PCA) was further applied to verify the risk model.

### Nomogram

The nomogram was created to better predict the survival by using the “RMS” packages in R. The concordance index and calibration plot were applied to test the reliability of the nomogram.

### The Investigation of the Immune Microenvironment

The tumor mutation burdens (TMBs) were evaluated and summed using the R package “maftools.” The CIBERSORT and ssGSEA algorithms were used to analyze the infiltration status of immune cells. In addition, we compared immune checkpoint activation in low-risk and high-risk patients using the “ggpubr” R package. Stromal score, immune score, and ESTIMATE score of patients were calculated using the “ESTIMATE” package to further explore the tumor microenvironment (TME) in LUAD patients.

### Exploration of Clinical Treatment

Using the R package “pRRophetic,” we evaluated their treatment responses according to half-maximal inhibition (IC_50_) per LUAD patient in terms of Cancer Drug Sensitivity (GDSC) (https://www.cancerrxgene.org/). The Tumor Immune Dysfunction and Exclusion (TIDE) algorithm was applied to explore the likelihood of the therapeutic immune response. The data of the immune subtype were downloaded on TIMER (http://timer.comp-genomics.org/) (Kong et al., 2021).

### Functional Analysis

The “clusterProfiler” package in R was conducted to carry out GO and KEGG enrichment analysis. GSEA analysis was performed to further screen functional pathways using GSEA 4.2.1 software (http://www.gesa-msigdb.org/gsea/index,jsp). Cytoscape (version 3.6.1) was used to establish the co-expression network between lncRNAs and mRNAs for visualization.

### Statistical Analysis

All statistical analyses and data visualization were conducted in R (https://www.r-project.org/, version 4.1.1). When no special instructions were given for the aforementioned methods of analysis, *p* < 0.05 was considered statistically significant.

## Results

### Screening the Oxidative Stress-Related lncRNAs in LUAD Patients

**FIGURE 1:**
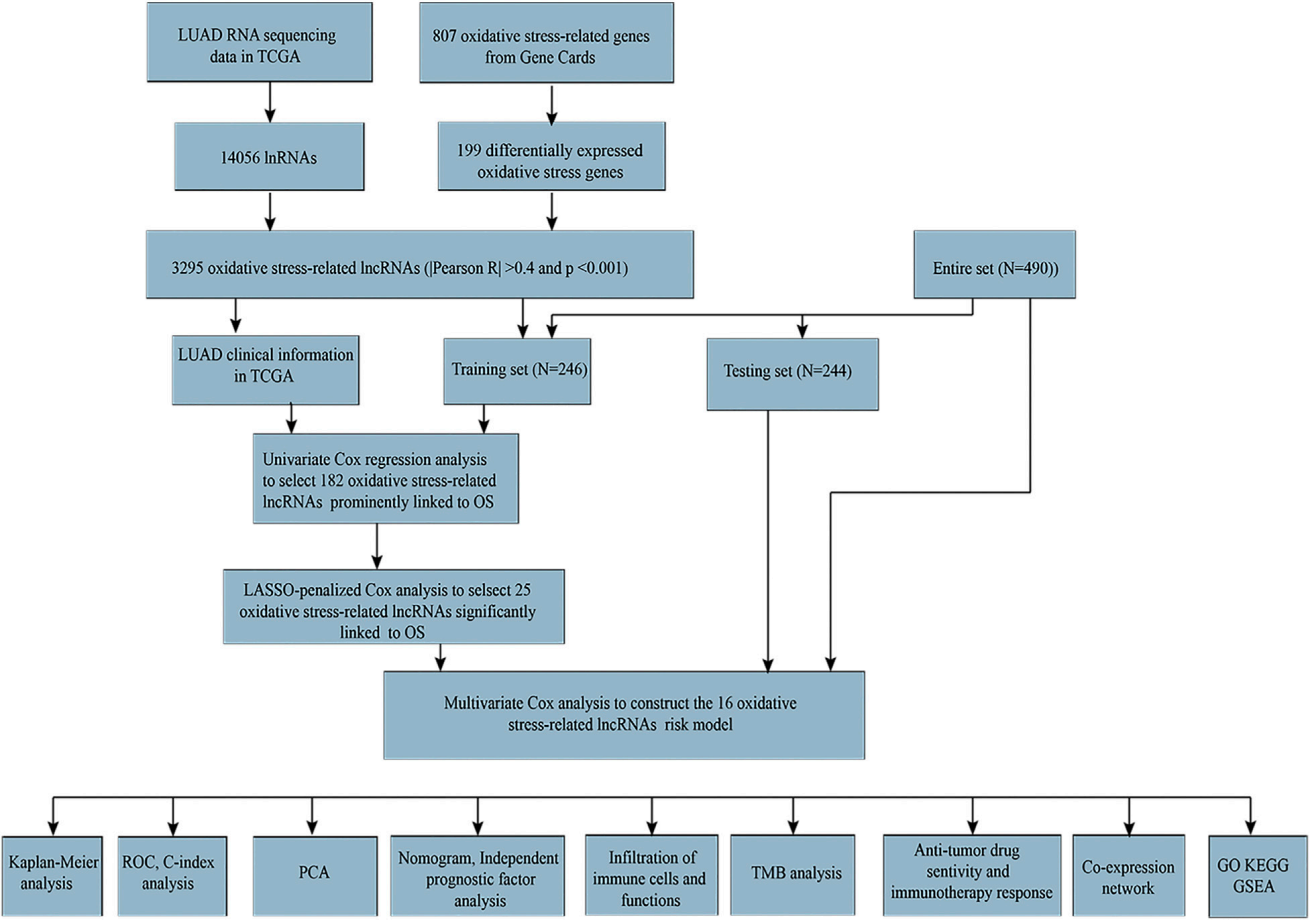
Flow diagram of complete data analysis.

**FIGURE 2:**
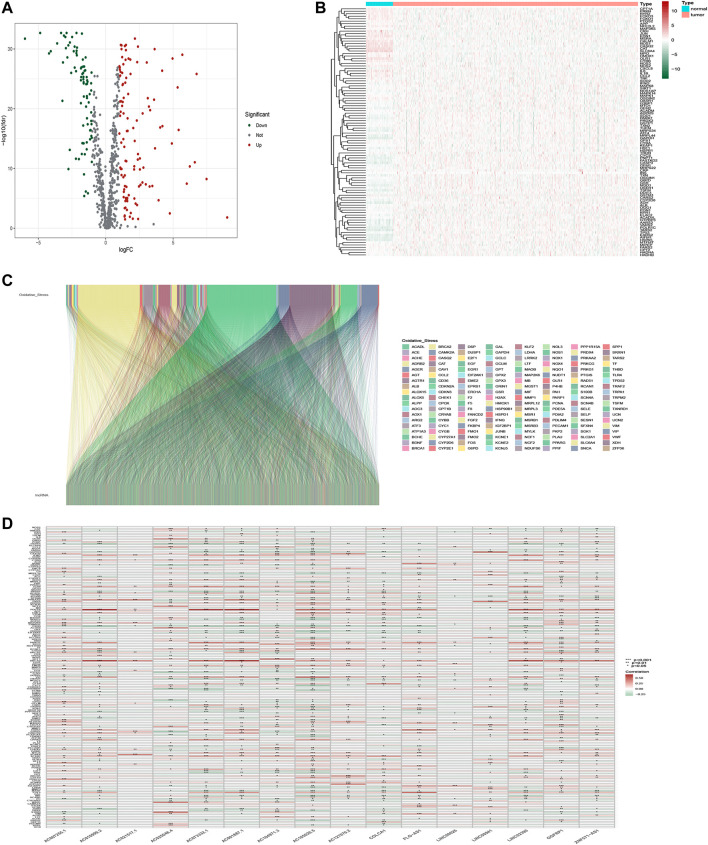
Identification of oxidative stress-related lncRNAs in LUAD patients. **(A)** Volcano plot of oxidative stress-associated DEGs in TCGA databases. **(B)**Heatmap of oxidative stress-associated DEGs in TCGA databases. **(C)** Sankey relation diagram for differentially expressed oxidative stress genes and oxidative stress-related lncRNAs. **(D)** Heatmap for the correlations between oxidative stress genes and oxidative stress-related lncRNAs.

The workflow is presented in Figure 1. Table 1 shows the clinical details of 490 patients with LUAD in the training and testing sets. In total, 807 oxidative stress-related genes were downloaded from GeneCards with a relevance score ≥7 (Supplementary Table S1). The expression of 199 differentially expressed oxidative stress genes was notably different among the normal samples and LUAD samples ((FDR) < 0.05 and |log2 fold change (FC)|≥1) (Supplementary Table S2). Among them, 115 were upregulated, and 84 were downregulated (Figure 2A), and a heatmap was drawn in Figure 2B. The oxidative stress–lncRNA co-expression network was shown in the Sankey diagram (Figure 2C), and 3,295 oxidative stress-related lncRNAs were discerned as oxidative stress-related lncRNAs (Pearson correlation coefficient >0.4, *p* < 0.001). The correlation between oxidative stress associated genes, like PDE5A and PRKG1, and lncRNAs were shown in (Supplementary Table S3), and displayed in (Figure 2D).

**TABLE 1:** Clinical details of 490 LUAD patients.

Covariate	Type	Total	Test	Train	*p-*value
Age	≤65	231 (47.14%)	125 (51.23%)	106 (43.09%)	0.0832
	>65	249 (50.82%)	114 (46.72%)	135 (54.88%)	
	Unknown	10 (2.04%)	5 (2.05%)	5 (2.03%)	
Gender	Female	262 (53.47%)	127 (52.05%)	135 (54.88%)	0.5912
	Male	228 (46.53%)	117 (47.95%)	111 (45.12%)	
Stage	Stage I–II	378 (77.14%)	191 (39.98%)	187 (38.16%)	0.7268
	Stage III–IV	104 (21.22%)	49 (10%)	55 (11.22%)	
	Unknown	8 (1.63%)	4 (1.64%)	4 (1.63%)	
T	T1–2	426 (86.94%)	210 (42.86%)	216 (44.08%)	0.7602
	T3–4	61 (12.45%)	32 (6.53%)	29 (5.92%)	
	Unknown	3 (0.61%)	2 (0.82%)	1 (0.41%)	
M	M0	324 (66.12%)	160 (65.57%)	164 (66.67%)	0.8098
	M1	24 (4.9%)	13 (5.33%)	11 (4.47%)	
	Unknown	142 (28.98%)	71 (29.1%)	71 (28.86%)	
N	N0	317 (64.69%)	165 (67.62%)	152 (61.79%)	0.5368
	N1–3	162 (33.06%)	74 (15.10%)	88 (17.96%)	
	Unknown	11 (2.24%)	5 (2.05%)	6 (2.44%)	

### Risk Model Construction and Validation

**FIGURE 3:**
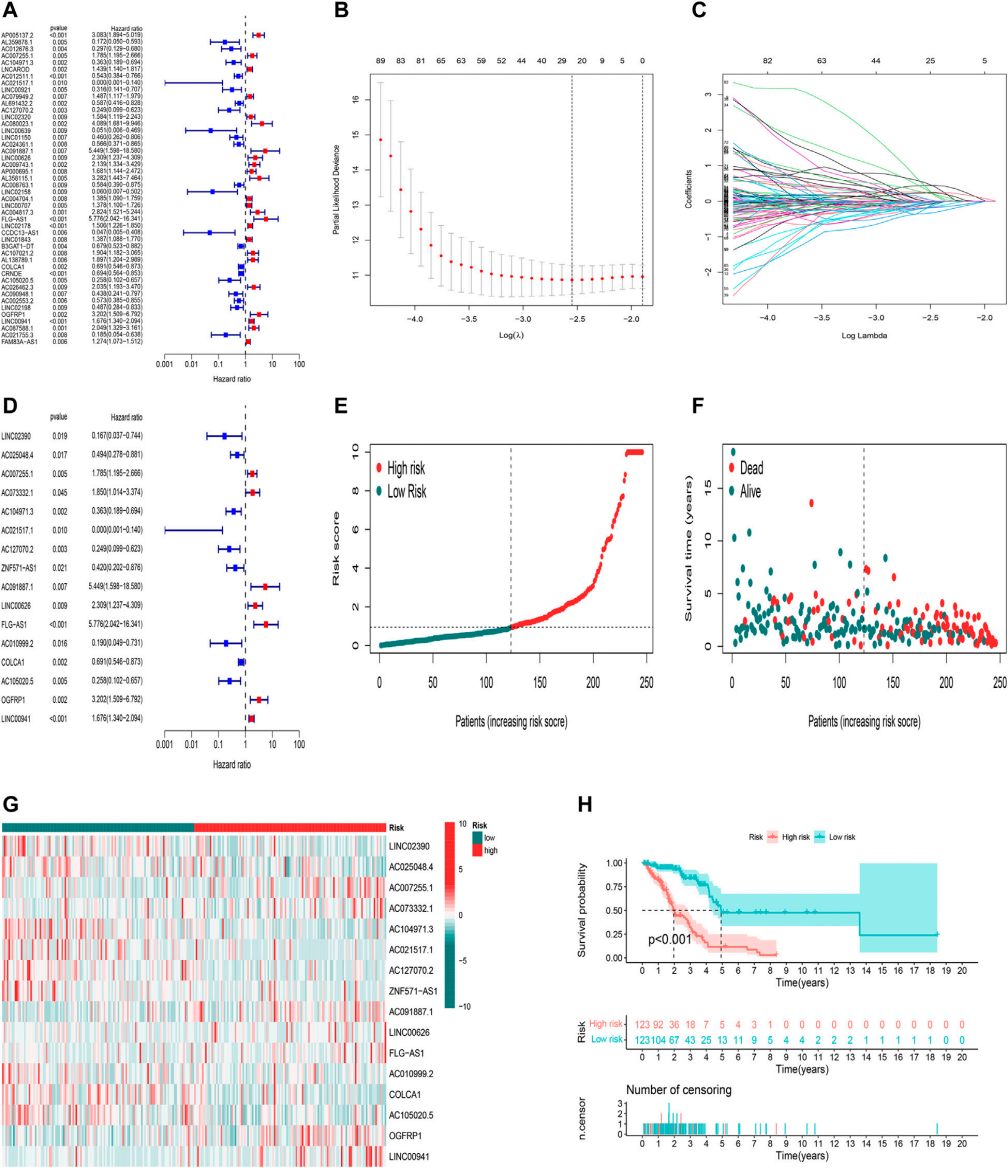
Construction and validation of the predictive model in TCGA training set. **(A)** Univariate Cox regression analysis of OS for part of 182 oxidative stress-related lncRNA prognostic signatures. **(B,C)** Altogether 25 lncRNAs were selected using LASSO regression. **(D)** Multivariate Cox regression analysis showed 16 independent prognostic lncRNAs. **(E)** Distribution of oxidative stress-related lncRNA model-based risk score for the training set. **(F)** Different patterns of survival status and survival time between high-risk and low-risk groups in the training set. **(G)** Heatmap to show the expression of 16 lncRNAs between high- and low-risk groups in the training set. **(H)** Kaplan–Meier curve of high-risk and low-risk patients in the training set.

Here, 182 oxidative stress-related lncRNAs were screened using univariate Cox regression analysis (Figure 3A, Supplementary Table S4). As a popular method, LASSO Cox analysis is widely used for the optimal selection of features from high-dimensional data. To prevent overfitting prognostic features, we used LASSO regression to analyze these lncRNAs, which identified 25 lncRNAs significantly associated with survival (Figures 3B,C). Finally, we used multivariate Cox regression analysis to identify the most powerful signatures. A total of 16 oxidative stress-related lncRNAs (Supplementary Table S5) were further identified and used to build a risk model (Figure 3D).

Calculation of the risk score is based on the following formula: risk score = expression of LINC02390×(-2.38547318096874)+ expression of AC025048.4×(-1.0744285140728)+ expression of AC007255.1×(0.642127461867371)+ expression of AC073332.1×(2.23226126557912)+ expression of AC104971.3×(-0.49082814485428)+ expression of AC021517.1×( -6.77265267596657)+ expression of AC127070.2×(-0.797790541274756)+ expression of ZNF571-AS1×( -1.24587634589269)+ expression of AC091887.1×(4.74804318521426)+ expression of LINC00626×(0.588193036671943)+ expression of FLG-AS1×(1.45742973729599)+ expression of AC010999.2×(-1.51189302537647)+ expression of COLCA1×(-0.266002934139891)+ expression of AC105020.5×(-1.01917959971841)+ expression of OGFRP1×(1.29144053714695)+ expression of LINC00941×(0.297685670482472).

Using the aforementioned signatures, we calculated the patient’s prognostic risk score. LUAD patients were divided into high-risk and low-risk groups following a median risk score. The distribution patterns of risk scores of LUAD patients between the high-risk and low-risk groups in the training set are shown in Figure 3E. Patients’ survival status and survival time in the high-risk and low-risk groups in the training set are described in Figure 3F. For each patient, the relative expression levels of 16 oxidative stress-related lncRNAs are presented in Figure 3G. K-M analysis showed that the low-risk group in the training set had more prolonged overall survival than the high-risk group (Figure 3H, *p*< 0.001).

**FIGURE 4:**
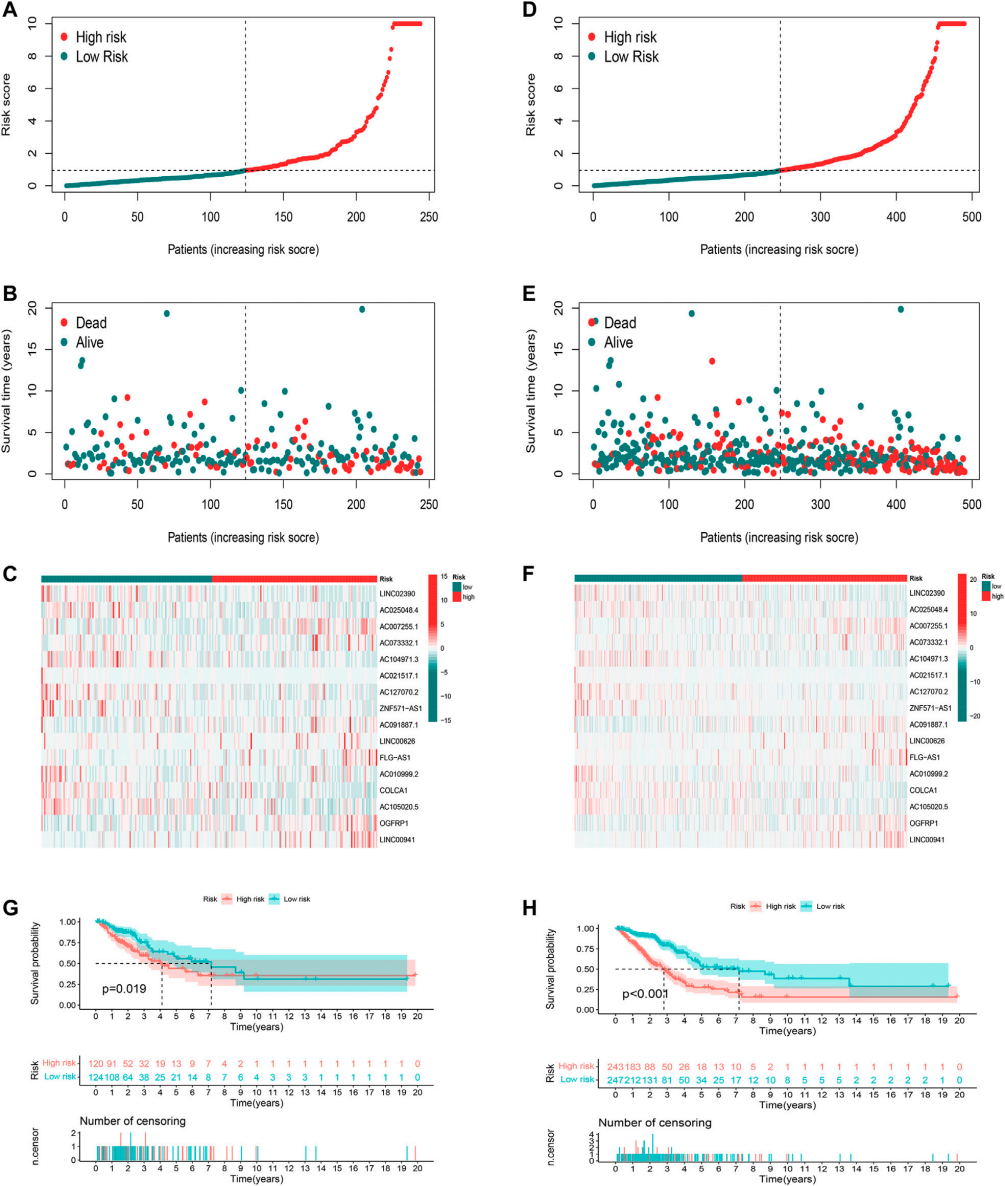
Validation of the prognostic oxidative stress-related lncRNA signature. **(A)** Risk score, **(B)** survival status, and **(C)** heatmap for the testing set. **(D)** Risk score, **(E)** survival status, and **(F)** heatmap for the entire set. **(G)** Kaplan–Meier curve for the testing set. **(H)** Kaplan–Meier curve for the entire set.

Using the uniform formula, we calculated risk scores for LUAD patients to validate the predictive capability of the established model. Figure 4 shows the diffusion of risk scores, survival status and time, and expression of the oxidative stress-related lncRNAs in the testing set (Figures 4A–C) and the entire set (Figures 4D–F). The K-M survival curve based on the testing set and the entire set also showed that the patients in the low-risk group had a longer OS than those in the high-risk group (Figures 4G,H, *p*<0.05).

### Nomogram and Independent Prognostic Factor Analysis

**FIGURE 5:**
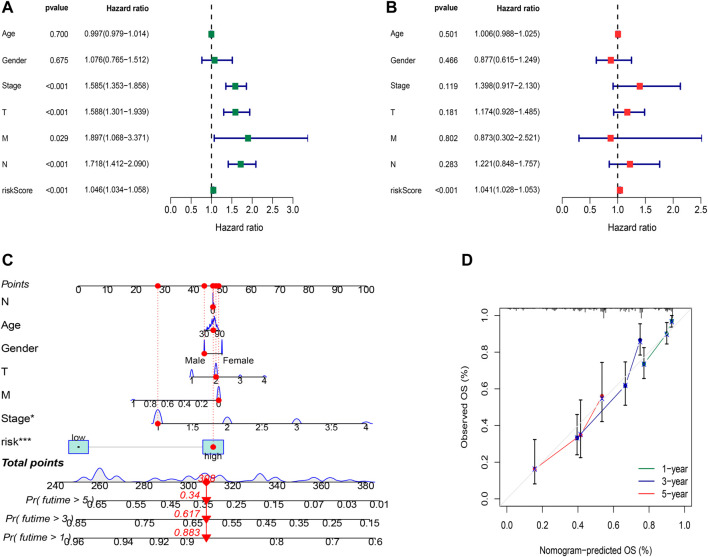
Independent prognostic factors and construction of the nomogram. **(A)** Univariate analysis of the clinical characteristic and risk score with the OS. **(B)** Multivariate analysis of the clinical characteristic and risk score with the OS. **(C)** Nomogram predicts the probability of the 1-, 3-, and 5-year OS. **(D)** Calibration plot of the nomogram indicates the probability of the 1-, 3-, and 5-year OS.

Whether the risk model can be used as an independent prognostic factor for LUAD was tested by applying univariate and multivariate Cox regression analyses. Univariate Cox regression analysis indicated that risk score, disease stage, and TNM stage were related to prognosis **(** Figure 5A, *p*<0.001**)**. Furthermore, multivariate Cox regression analysis presented that the risk score was an independent factor affecting prognosis (Figure 5B, *p*<0.001). According to the aforementioned results, it was concluded that the risk model based on the 16 oxidative stress-related lncRNAs had a significant impact on the survival and prognosis of LUAD patients and were independent prognostic factors.

To better predict the 1-,3-,5-year survival for LUAD patients, we established a nomogram combining gender, age, stage, TNM and risk score (Figure 5C). Using calibration curve analysis, the prediction accuracy of the nomogram was assessed (Figure 5D).

### Assessment of the Risk Model

**FIGURE 6:**
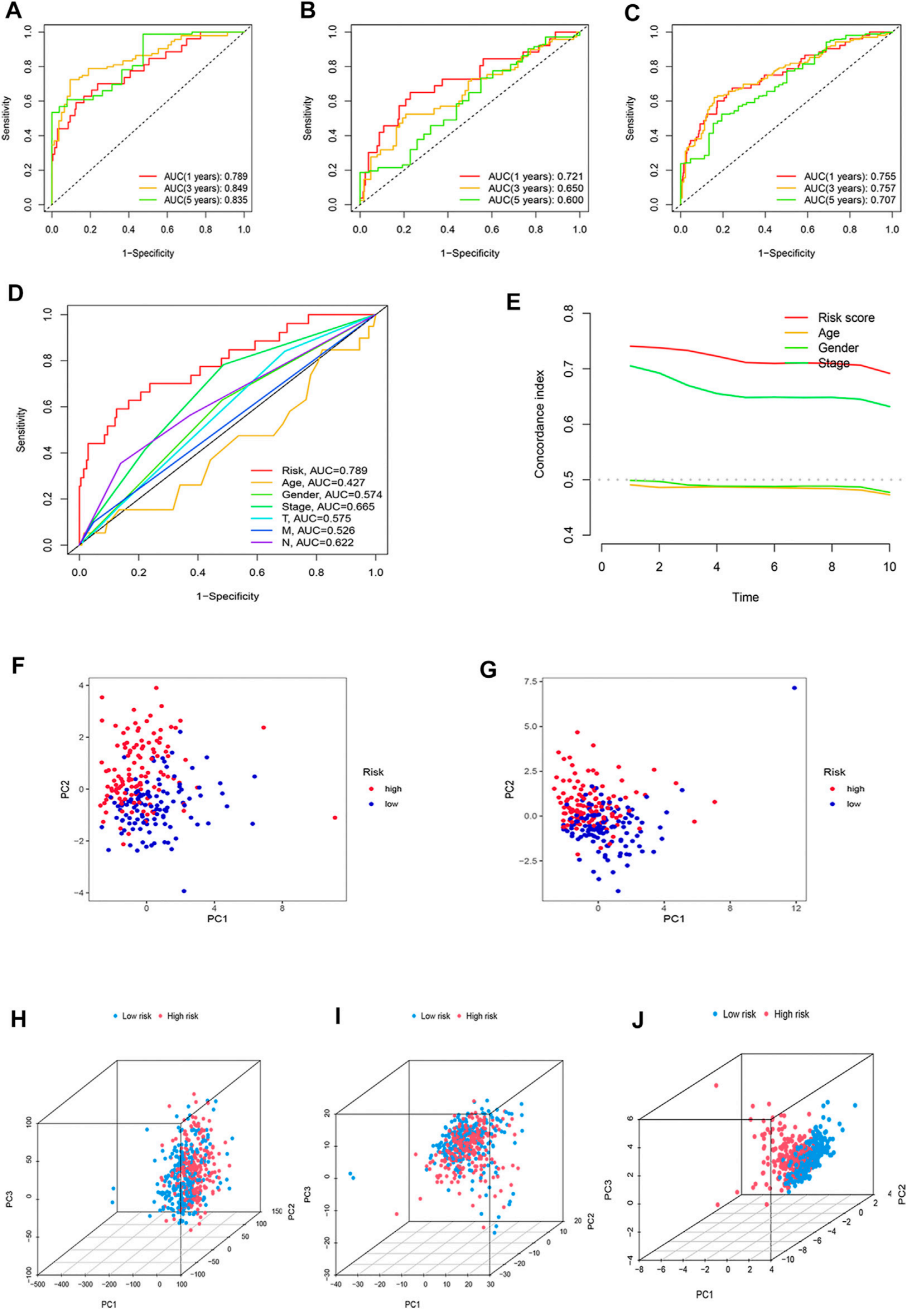
Assessment of the predictive risk model and principal component analysis. The 1-, 3-, and 5-year ROC curves of the **(A)** training set, **(B)** testing set, and **(C)** entire set. **(D)** ROC curves of the clinical characteristics and risk score. **(E)** Concordance indexes of the risk score and clinical features. **(F)** PCA between high-risk and low-risk groups based on 16 prognostic lncRNAs in the training set **(G)** and testing set. **(H)** PCA between the high-risk and low-risk groups based on entire gene expression profiles, **(I)** all oxidative stress genes, **(J)** and risk model based on the representation profiles of the 16 oxidative stress-related lncRNAs in the entire set.

The sensitivity of the risk model was evaluated using time-dependent receiver operating characteristics (ROCs). The 1-, 3-, and 5-year AUC of the training set was 0.789, 0.849, and 0.835, while in the testing set, they were 0.721, 0.650, and 0.600, and of the entire set were 0.755, 0.757, and 0.707, respectively (Figures 6A–C). The AUC of the risk model was significantly higher than that of other clinicopathological features, indicating that the 16 oxidative stress-related lncRNAs are relatively reliable in the prognostic risk model of LUAD (Figure 6D). The concordance index also showed the accuracy of the risk model (Figure 6E). To further assess the group ability of the oxidative stress-related lncRNA model, we applied principal component analysis (PCA) to test for differences between high-risk and low-risk groups (Figures 6F,G). Additionally, we used PCA to verify the authenticity of the risk model constructed based on the complete gene expression profile, 199 oxidative stress-related differentially expressed genes, and risk model sorted by the expression of the 16 oxidative stress-related lncRNAs (Figures 6H–J). The results suggested that the risk model based on oxidative stress-related lncRNAs was able to distinguish high-risk and low-risk groups of patients.

**FIGURE 7:**
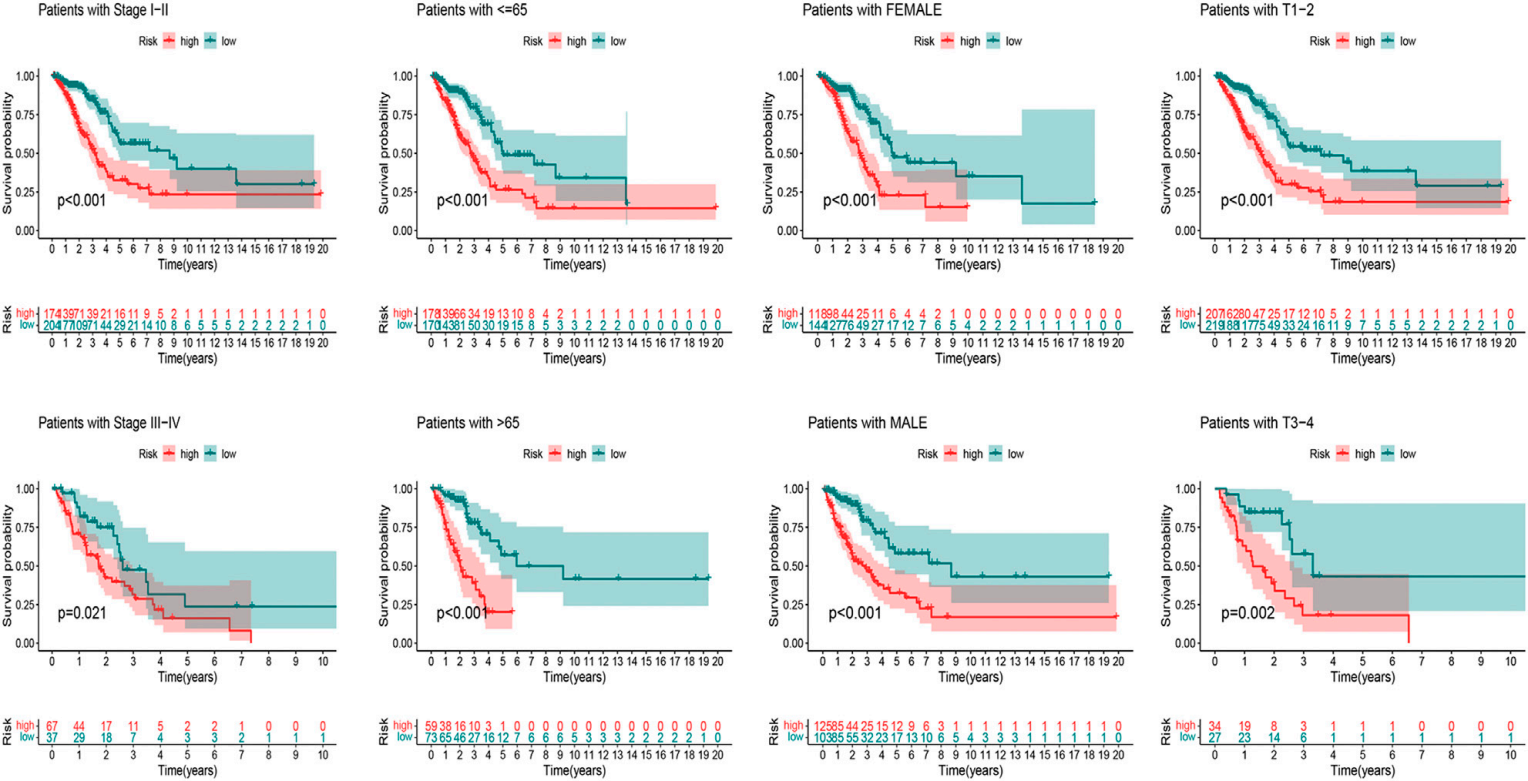
Kaplan–Meier curves of OS difference stratified by LUAD stage (I–II or III–IV), age (≤65 or >65), gender (female or male), and TNM stage (T1–2 or T3–4) between high-risk and low-risk groups in TCGA entire set.

According to the universal clinicopathological characteristics, we evaluated the discrepancies of LUAD patients between the low-risk and high-risk groups. By dividing patients into groups based on gender, age, stage, or TNM, results indicated that the OS of the patients in the low-risk group was longer than that in the high-risk group (Figure 7).

### Stratification Analysis of the Oxidative Stress-Related lncRNA in Immune Features

**FIGURE 8:**
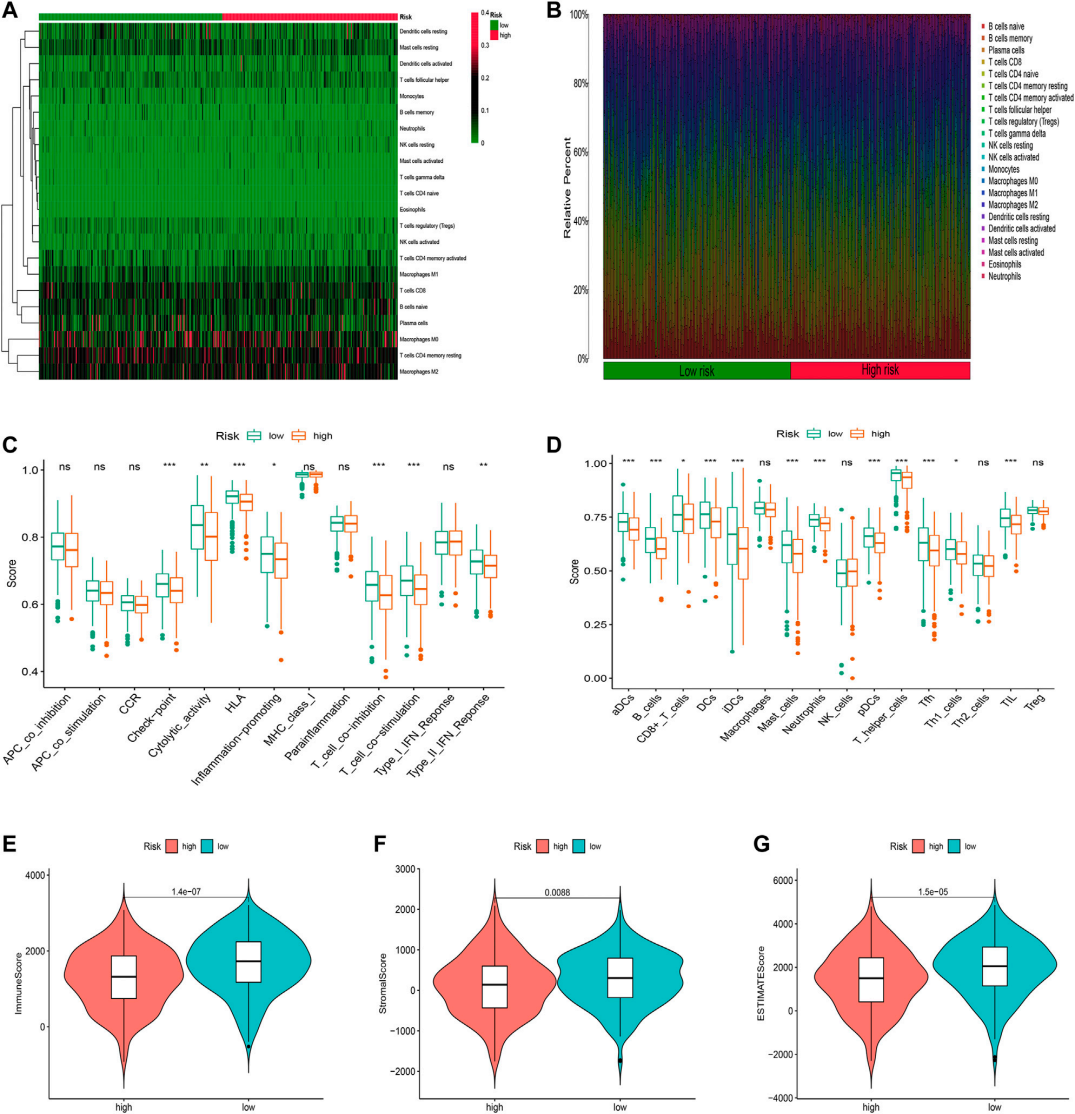
Immune infiltration discrepancy in different risk groups. **(A)** Heatmap of 22 tumor-infiltrating immune cell types in low- and high-risk groups. **(B)** Bar chart of the proportions for 22 immune cell types. **(C)** ssGSEA scores of immune functions in low-risk and high-risk groups. **(D)** Immune cells in low-risk and high-risk groups. **(E–G)** TME scores between high- and low-risk groups. *p < 0.5, **p < 0.01, and ***p < 0.001; ns, no sense.

We first used the CIBERSORT algorithm to explore patients’ immune cell infiltration status in the high-risk and low-risk groups (Supplementary Table S6). Figures 8A,B exhibited the fractions of 22 kinds of immune cells in high-risk and low-risk groups. We further assessed the relative abundance of 22 tumor-infiltrating immune cells in each patient to investigate better the underlying molecular mechanisms of oxidative stress-related lncRNAs and their correlations with tumor immunity using the ssGSEA algorithm (Supplementary Table S7). The results showed that many immune cells and immune responses were related to the risk score. The immune functions like Check−point, T_cell_co−inhibition, and Type_II_IFN_Reponse were higher in the low-risk group (Figure 8C). The infiltration of aDCs, B_cells, DCs, iDCs, neutrophils, T_helper_cells, Tfh, and TIL was significantly higher in the low-risk group (Figure 8D). Furthermore, we found that LUAD patients in the low-risk group had substantially higher stromal, immune, and ESTIMATE scores, suggesting that the TME was different from the high-risk group (Figures 8E–G).

### Somatic Mutation Landscape

**FIGURE 9:**
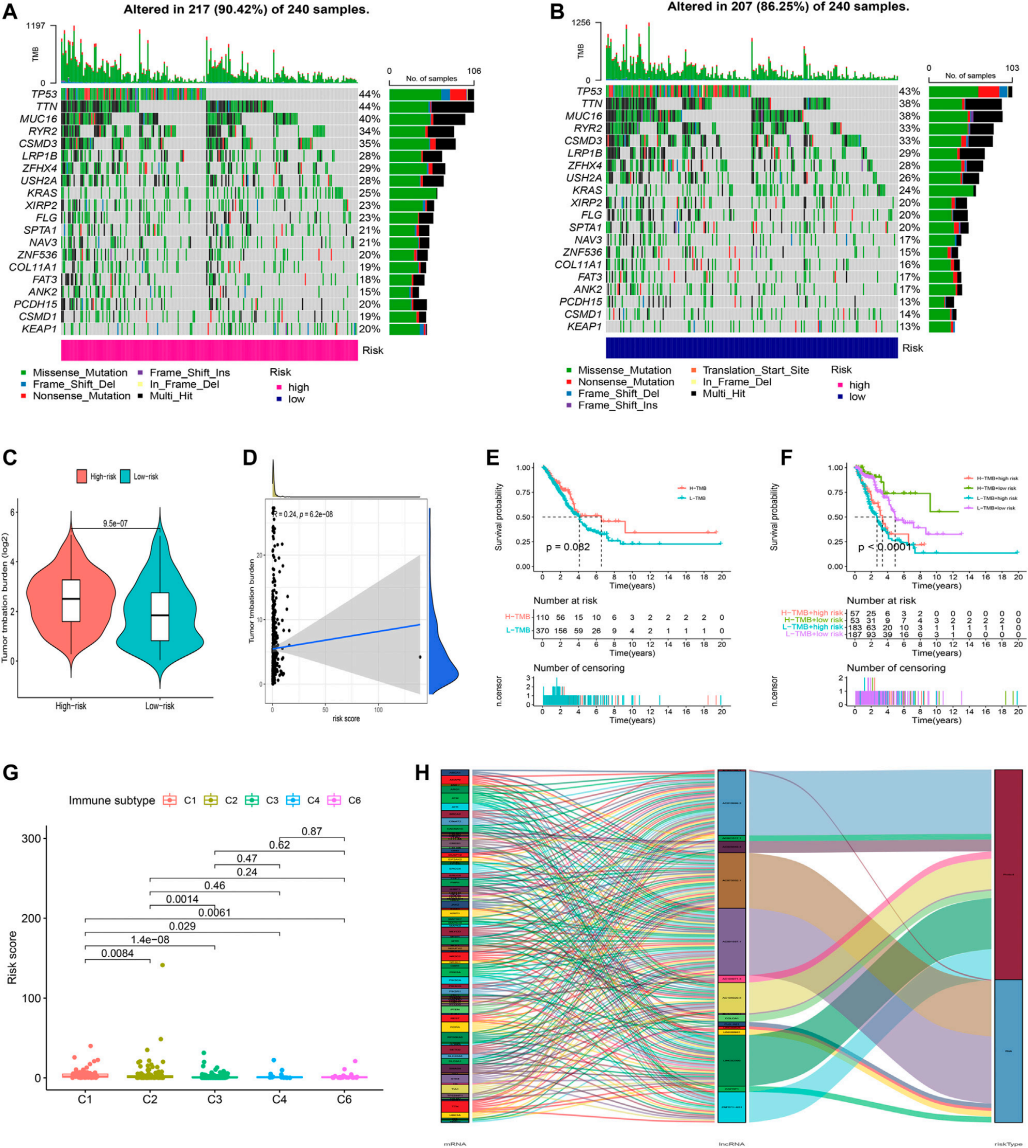
Exploration of tumor mutation burden and visualization of lncRNA networks. **(A,B)** Waterfall plot of somatic mutation features established with high- and low-risk groups. **(C)** Tumor mutation burden in the high-risk and low-risk groups. **(D)** Correlation between risk score and TMB. **(E)** Kaplan–Meier curve of the OS among the high- and low-TMB groups. **(F)** Survival analysis among four patient groups stratified by both TMB and risk score. **(G)** Correlation between the risk score and immune subtype. **(H)** Connection degree between the oxidative stress-related lncRNAs, oxidative stress-related genes, and risk types.

Further analysis of the mutational landscape of somatic cells was conducted in LUAD patients. Based on the comparison, approximately 90.42% of patients exhibited genetic mutations in high-risk patients, while 86.25% of samples exhibited genetic mutations in low-risk samples (Figures 9A,B). The TMB of patients in the high-risk group was significantly higher than that in the low-risk group (Figure 9C, *p*<0.05). Therefore, we tested the correlation between the risk model-based oxidative stress-related lncRNAs and TMB using Spearman correlation analysis (Figure 9D, *R* = 0.24, *p* = 6.2e-08). The results suggested a strong correlation between the oxidative stress-based classifier index and the TMB. To investigate the impact of TMB state on prognosis in LUAD patients, we applied survival analysis based on high and low TMB groups. However, the survival curve of patients with high TMB was similar to that of patients with low TMB, indicating that the TMB failed to distinguish the survival in LUAD (Figure 9E, *p*>0.05). Moreover, we tested whether the TMB-combined risk score could accurately predict the OS outcome, as shown in Figure 9F, *p*<0.05. The results showed that the oxidative stress-related lncRNA model has better prognostic significance than the single tumor mutation burden status.

In addition, according to the immune subtype data from TIMER2.0 (Supplementary Table S8), we tested whether the risk model based on the 16 oxidative stress-related lncRNAs could distinguish the different immune subtypes (Figure 9G). The result suggested that the risk model had a high discriminative power with the immune subtype. Furthermore, oxidative stress genes, 16 oxidative stress-related lncRNAs, and risk types were included in the Sankey network (Figure 9H). These results may provide some insights into the role of oxidative stress–lncRNAs in LUAD oncogenesis.

### Clinical Treatment and Drug Sensitivity Analysis

**FIGURE 10:**
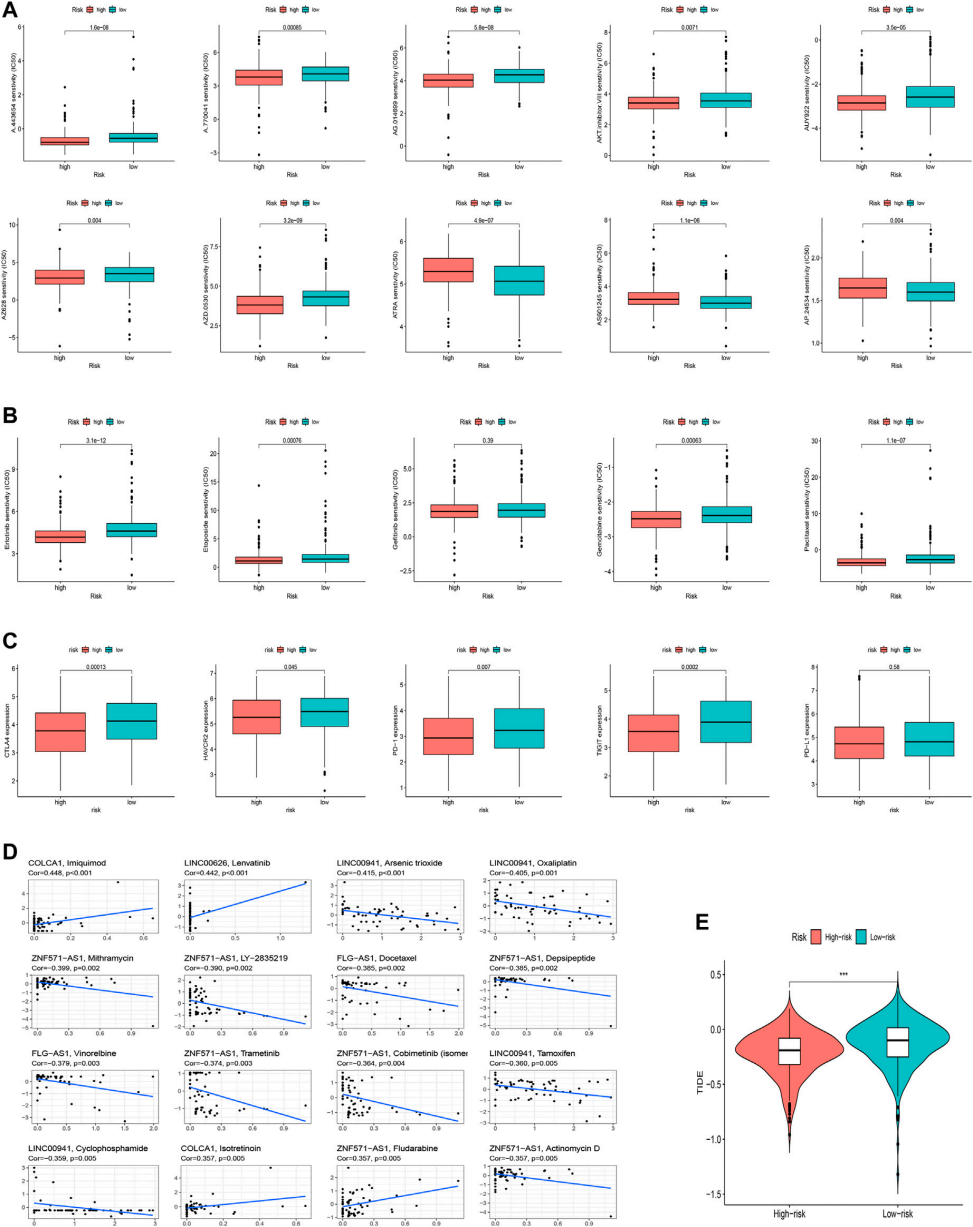
Clinical application of the risk signature. **(A)** Comparison of IC_50_ of chemotherapeutic drugs among two subgroups. **(B)** Investigation of anti-tumor drug sensitivity-targeting signature. **(C)** Expression levels of CTLA4, HAVCR2, PD-1, TIGIT, and PD-L1 in the high- and low-risk groups. **(D)** Correlation between 16 lncRNAs and chemotherapeutic drugs. **(E)** TIDE prediction difference in the high-risk and low-risk groups.

We speculated that patients in the high-risk and low-risk groups might have different responses to drugs, chemotherapy, critical ICPs, and immunotherapy because of the different immune microenvironments between the two groups. Therefore, to test our hypothesis, we used the R package “pRRophetic” to assess treatment response according to the half-maximal inhibitory concentration (IC_50_) available in the GDSC database for LUAD patients. The IC_50_s of A.443654, A.770041, AG.014699, AUY922, AKTinhibitors VIII, AZ628, and AZD.0530 were significantly higher in the low-risk group (Figure 10A), indicating that exposure to these drugs might be more appropriate for high-risk patients. Additionally, we counted the IC_50_ of common anti-lung cancer drugs in two subgroups. Patients in the low-risk groups were related with a higher IC_50_ of targeted therapy such as erlotinib (*p* < 0.05) and gefitinib (*p* > 0.05) and chemotherapeutics like paclitaxel (*p* < 0.05), etoposide (*p* < 0.05) and gemcitabine (*p* < 0.05), which indicated that the risk model served as a promising predictor of anti-tumor drug sensitivity (Figure 10B). In addition, with ICIs have been applied in the treatment of LUAD and other cancers, we further explored the differences in ICI-related biomarker expression among two subgroups. The results presented that the low-risk group had high CTLA4 (*p* < 0.05), HAVCR2 (*p* < 0.05), PD−1 (*p* < 0.05), TIGIT (*p* < 0.05), and PD-L1 (*p* > 0.05) expression (Figure 10C). Furthermore, we analyzed the sensitivity between hub oxidative stress-lncRNAs and drugs (Supplementary Table S9). For example, the highest correlation coefficient is between imiquimod and COLCA1 (Figure 10D, Cor = 0.448 *p*<0.001). Our study suggested that we could select appropriate drugs based on risk regrouping among LUAD patients.

We finally explored the correlation between oxidative stress-related lncRNAs and immunotherapy-related indicators. Similarly, we found that the low-risk group was more sensitive to immunotherapy than the high-risk group, suggesting that this oxidative stress-based classification index can be used as a predictor of TIDE (Figure 10E, *p*<0.001).

### Functional Analysis

**FIGURE 11:**
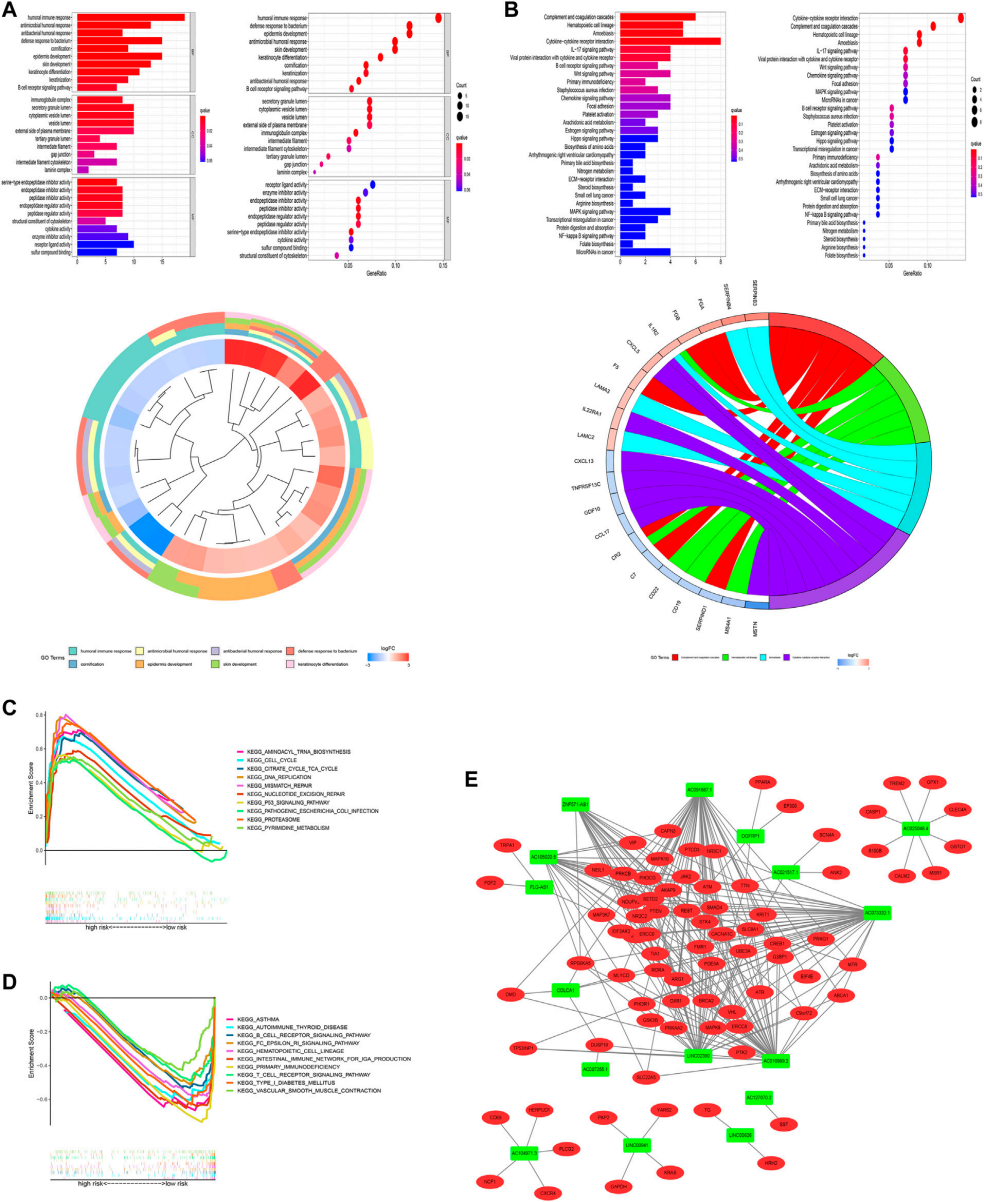
Functional analysis. **(A)** Top 10 classes of GO enrichment terms based on DEGs between two groups, including biological process (BP), cellular component (CC), and molecular function (MF). **(B)** Top 30 pathways of KEGG enrichment terms. **(C)** Gene set enrichment analysis of the top 10 pathways significantly enriched in the high-risk group. **(D)** Gene set enrichment analysis of the top 10 pathways enriched considerably in the low-risk group. **(E)** Cytoscape of lncRNA–mRNA co-expression network. Green nodes represent lncRNAs, while red nodes represent mRNAs.

Given that the current study has not fully elucidated the mechanism of occurrence and progression of LUAD, we performed a functional enrichment analysis of differentially expressed genes (DEGs) between high-risk and low-risk groups ((|log2-fold change (FC)| ≥ 1, *p* < 0.05)). As shown in Figure 11A, Supplementary Table S10, GO enrichment analysis indicated that they mainly participate in the modulation of humoral immune response, immunoglobulin complex, and serine-type endopeptidase inhibitor activity, and so on. KEGG enrichment analysis presented that these lncRNAs were primarily connected with complement and coagulation cascades and hematopoietic cells (Figure 11B). We further explored the differences in biological functions between high-risk and low-risk groups using GSEA software (Figures 11C,D, Supplementary Table S11). Pathways such as aminoacyl tRNA biosynthesis and cell cycle were significantly enriched in the high-risk group. Still, pathways like asthma and autoimmune thyroid disease were highly enriched in the low-risk group. The results of these functional enrichment analyses fully demonstrate the unusual close management between LUAD and the immune system and provide support for our exploration of immunotherapy for LUAD. In addition, to explore how the key lncRNAs we screened affect the LUAD process by affecting differential genes, we constructed an lncRNA–mRNA interaction network (Figure 11E).

## Discussion

Lung adenocarcinoma is the most common type of lung cancer and is distinguished from other lung tumors by its unique cellular and molecular features (Zappa and Mousa, 2016; Sainz de Aja et al., 2021). LUAD has a high degree of malignancy and a lack of early diagnosis methods, which also leads to an inferior prognosis of LUAD, that is, the 5-year survival rate of patients is often less than 15% (Spella and Stathopoulos, 2021; Šutić et al., 2021). Therefore, a deeper understanding of the occurrence and development mechanism of LUAD and the search for more accurate diagnostic and prognostic biomarkers are of great significance.

Oxidative stress is a pathological response in organisms, which means an imbalance between the production and consumption of ROS (Flohé, 2020). With the deepening of research, oxidative stress has been found to serve in the process of various diseases (Badanjak et al., 2021; Forman and Zhang, 2021; Kyriazis et al., 2021). Several recent studies have also pointed out the role of oxidative stress in LUAD. For example, the survey by Galan-Cobo et al. (2019) showed that deletion of the *LKB1* gene in LUAD led to activation of the KLK pathway, ultimately leading to increased oxidative stress in the corresponding cells. Similarly, Hu et al. (2020) also pointed out that inhibition of the SLC7A11/glutathione axis significantly prolonged the survival time of KRAS-mutant LUAD mice. It can be seen that oxidative stress is of great significance in the process of LUAD. It is worth noting that although there have been experiments using oxidative stress-related indicators as clinical markers (Skoulidis et al., 2015; Sharma and Kanwar, 2018), most of them target specific LUAD subspecies, and there is still a lack of oxidative stress-related biomarkers for generalized LUAD, while lncRNA-based markers are even rarer.

In our study, 16 lncRNAs related to oxidative stress were selected to construct risk models. Most of them have no relevant research at present. With the help of bioinformatics methods, ZNF571-AS1 has been shown to predict the prognosis of dilated cardiomyopathy and acute myeloid leukemia (Pan et al., 2017; Chen et al., 2021). FLG-AS1 was powerfully demonstrated to predict pathological outcomes after therapeutic intervention in esophageal squamous cell carcinoma (Zhang et al., 2020). Similarly, AC025048.4 and AC007255.1 have demonstrated diagnostic and prognostic values in lung adenocarcinoma and esophageal carcinoma, respectively (Wang et al., 2021; Zheng Z. et al., 2021). However, there are many related studies on OGFRP1, LINC00941, and COLCA1. Recent studies have demonstrated that lncRNA OGFRP1 may promote tumor progression by regulating metabolism or mediating endothelial–mesenchymal transition in tumors of the digestive system and female reproductive system (Zou et al., 2019; Zhang et al., 2021; Dong et al., 2022). The study of Xiaojing Liu et al. also pointed out that this lncRNA can play a tumor-promoting role in non-small cell lung cancer through the miR-4640-5p/eIF5A axis. In addition, in our follow-up immune cell-related abundance analysis, it was found that the plasma B cell abundance level in the high-risk group was higher, which was consistent with the study of Zhou et al. (2021), which suggested that the increased expression of OGFRP1 may be one of the reasons for the poor prognosis of patients in the high-risk group of LUAD and may become a focus of tumor immunotherapy-targeting B cells. Similarly, LINC00941 has also been shown to be closely related to digestive system tumors in multiple studies. It can affect classic cancer-related pathways or genes such as the WNT/β-catenin pathway and MYC gene through specific regulatory axes (Ai et al., 2020; Chang et al., 2021; Wu N. et al., 2021). While COLCA1 was first thought to be related to the susceptibility of colorectal cancer (Peltekova et al., 2014), recent studies have pointed out its relationship with the exposure of primary biliary cholangitis (Hitomi et al., 2021). As for the survey by Zheng J. et al. (2021), there was a close relationship between the level of COLCA1 N-6 methylation and the tumor microenvironment of lung adenocarcinoma. In addition, we also noticed that among all 16 lncRNAs, AC021517.1 has the highest absolute value of the coef coefficient but was rarely studied, which may indicate that this RNA may play a vital role in the LUAD effect.

Interestingly, in the drug sensitivity correlation analysis, we found that COLCA1, FLG-AS1, LINC00941, OGFRP1, and ZNF571-AS1 lncRNAs showed statistically significant correlations with multiple drugs. Among them, LINC00941 showed the broadest correlation (related to the sensitivity of 25 drugs), and COLCA1 showed the highest positive correlation with imiquimod (Cor = 0.448 *p* < 0.001). Imiquimod is a Toll-like receptor 7 (TLR-7) activator, which can activate innate immune cells via TLR-7 or induce apoptosis and autophagy in cancer alone (Edwards, 2000; Huang et al., 2016). The study by Chuang et al. (2020) found that imiquimod can induce severe ROS production in skin cancer cells, which is consistent with our results, implying that oxidative stress may be a solution to the high chemoresistance of LUAD a potential entrance.

In addition, the functional enrichment analysis indicated that those DEGs between high-risk and low-risk groups were strongly correlated with human immune responses. According to ssGSEA analysis, T helper cells (Th cells) and HLA (human leukocyte antigen) systems were highly related to the risk score. Also, the results of immune correlation analysis pointed out that the high-risk group had more obvious immunosuppression than the low-risk group, and the scores of T helper cells and HLA were also lower (Zhu, 2018; Dong, 2021). The cells transform into different phenotypes after receiving other inflammatory stimuli. Recent studies have shown that in systemic lupus erythematosus, oxidative stress can shift Th cells toward pathogenic Th17 (Ohl and Tenbrock, 2021); similarly, vancomycin-induced gut oxidative stress can induce a Th1/Th17 bias in the Th-cell population in patients with colitis (Strati et al., 2021). ROS is necessary for the fate of Th cells (Franchina et al., 2018). Unsurprisingly, the latest study by Dejima et al. (2021) pointed out that the reduced infiltration of Th cells may be a key factor leading to the early carcinogenesis of LUAD. Clinical studies by Guo et al. (2017) also showed that compared with lung squamous cell carcinoma, LUAD patients had low levels of circulating Th cells. All of the aforementioned evidence points to the unique potential of Th cells in the treatment and prognosis of LUAD. The HLA system is critical in mediating immune defense, distinguishing between self and foreign cells to direct the target of immune killing (de Bakker et al., 2006; Redwood et al., 2018). HLA has numerous alleles, and different allelic variants lead to different binding specificities of HLA proteins (Jeiziner et al., 2021). It is worth noting that the HLA system has also been found to play a significant role in tumors, not limited to allergic reactions and rejection reactions. For example, HLA-G molecules exist at high levels in the tumor environment and have excellent potential to become immune checkpoint therapy (Attia et al., 2020; Loustau et al., 2020); the silent mutation or deletion of HLA molecules has proved to be a relatively common phenomenon in cancer (Shukla et al., 2015). The loss of HLA also occurs in LUAD, and the proportion of occurrence is not low (Dejima et al., 2021). But at the same time, we also noticed that the research of van de Water et al. (2021) showed that the expression level of HLA-G was inconsistent with the prognosis of lung cancer, but the prognosis and HLA-G level of patients with breast cancer, esophageal cancer, gastric cancer, and hepatocyte maintained a good correlation. This is in common with our findings and contradicts them, implying that the mutational diversity of HLA molecules has the value for further study.

Of course, there are some limitations to this study. First, this study was based on bioinformatics technology, and the database limited the reliability and applicability of the results, so some animal experiments or cell experiments need to be supplemented in the future; second, the oxidative stress-related data set we used was based on previous research results, so there may be some one-sidedness.

Here, we conducted the first integrated study of LUAD patients to reveal the relationships between the oxidative stress-related lncRNAs and LUAD. The expression profiles of lncRNAs and oxidative stress genes were identified, and a prognosis prediction model and a nomogram were built based on oxidative stress-related lncRNAs. Functional analysis and drug sensitivity analysis depending on the oxidative stress-related lncRNA signature were also performed. Additionally, we explored the relationship association with immunotherapy responses. All these studies aim to examine the role of oxidative stress in LUAD patients and provide new ideas for the precise treatment of LUAD in the future.

## Data Availability

The original contributions presented in the study are included in the article/Supplementary Material; further inquiries can be directed to the corresponding authors.
